# Simulation for Teaching on Racial Microaggressions and Bystander Intervention — A Theory-Based Guide for Health Profession Education

**DOI:** 10.1007/s40670-023-01820-0

**Published:** 2023-07-03

**Authors:** T. Dada, W. Laughey

**Affiliations:** 1 Health Professions Education Unit, York Medical School, University of Hull, Hull, YO10 5DD UK; 2 University Hospitals of Leicester NHS Trust, Leicester, UK

**Keywords:** Microaggressions, Simulation, Bystander training, Inequalities

## Abstract

Microaggressions are words or behaviour that “subtly and unconsciously express a prejudiced attitude”, and racial microaggressions express these attitudes towards people from racial minority groups. The “Bystander Effect” is when the presence of other people means that an individual is less likely to offer assistance or get involved in a situation — bystander intervention training aims to inform about the best ways to avoid this, equipping students with the necessary strategies. In health profession education, teaching on microaggressions and bystander intervention can be done with the use of simulation. Simulated patients (SPs) and environments would be the most appropriate modality of simulation to use, as roleplay would be central. This guide focuses on how to use simulation for teaching on racial microaggressions and bystander training for healthcare students including tips on preparing the students and SPs, planning for the implementation of the simulation training, setting ground rules, showing different scenarios, checking student understanding throughout, using debriefs and course evaluation feedback, and signposting students to available support afterwards. These are topics which are particularly relevant because there have been calls in recent years for healthcare education to be more inclusive and representative of current issues, as the COVID-19 pandemic and resurgence of the Black Lives Matter movement have highlighted curriculum gaps. So teaching students about this early is a good start, and simulation is an effective teaching method to help with this.

Imagine this: During a bedside teaching session for medical students, a patient asks to call the students who aren’t Caucasian “Bob” and “Jim” because it’s “easier”. The patient then goes on to comment on how good their English is and how “articulate” they are. As the tutor leading this session, what would you do?

This scenario depicts two examples of microaggressions, which are words or behaviours that “subtly and unconsciously express a prejudiced attitude;” racial microaggressions are those that express these attitudes towards people from racial minority groups [1]. Going back to the scenario at the start, the first microaggression here is an invalidation of the students’ identities by asking to address them by different names, whilst the second microaggression is a passive aggressive ascription of intelligence — the assumption that a non-Caucasian student is not as intelligent or as able to speak English as a Caucasian one.

Although most of the time these comments and actions are not said or done with the intention of causing offencee, they can still have a significant effect — it has been shown that continuous microaggressions can have adverse effects on mental health, increasing risks of stress, anxiety and depression [2].

The “Bystander Effect” is when the presence of other people means that an individual is less likely to offer assistance [3]. It has been widely studied and often ascribed to diffusion of responsibility: as the number of bystanders increases in a situation, the feeling of personal responsibility decreases [4]. Bystander intervention training teaches how to avoid being a bystander and provides the necessary strategies to accomplish this.

In undergraduate health professions education (HPE), teaching on microaggressions and bystander intervention can be achieved through simulation. A benefit of using simulation is the provision of more learning opportunities for students, as it enables them to practise their interventions and communication skills. Simulation can also help healthcare professionals become better prepared for unexpected events [5], and racial microaggressions are one example of unexpected events occurring in health settings. In this guide, the terms “simulated patients”, “SPs”, and “actors” will be used interchangeably to refer to the roleplayers.

The use of roleplay in teaching about microaggressions in general is not a new concept, having been successfully utilised by Koch et al. [6] to “increase student knowledge and comfort” in caring for transgender patients. Seventy-two nursing students role-played simulated scenarios that encouraged “culturally sensitive” patient assessments and supported discussions around the importance of speaking up to confront insensitive behaviour; the roleplays also encouraged a patient centred approach to support and communicate better with transgender patients. After the simulation, the nursing students reported feeling more comfortable and confident in advocating and communicating with transgender patients, their families, and also their fellow colleagues [6].

Similarly, Fisher et al. [7] included roleplays in a workshop for internal medicine residents as part of a microaggression response toolkit (MRT). The workshops lasted 50 minutes, and surveys were given before and after to the 85 residents who took part, to assess the usefulness of the workshops. There was an overall improvement in self-reported confidence and comfort with identifying, understanding, and responding to microaggressions.

This guide will focus on how to use simulation for teaching on racial microaggressions and bystander training in health professions education, particularly relevant following the Black Lives Matter (BLM) protests of 2020 — the death of George Floyd sparked a resurgence of the BLM movement, sparking global debate on systemic and institutional racism, and shining a spotlight on the need for institutions to educate about and combat this, including in HPE [8].

Despite this, there is relatively little direct teaching on racial microaggression, prompting calls for more attention to this area [9]. There is a clear requirement for more “anti-racist” teaching, confronting the historical and structural roots of healthcare inequality. The combination of such teaching with roleplay is effective in reducing prejudice in students [10]. Conscious and unconscious biases can present as both macro- and microaggressions, and these lie at the root of inequality. A good place to start is education on how to recognise racial microaggressions and how to challenge them, as this improves outcomes for ethnic minority students and clinicians, creating a more “inclusive environment” [11].

Here, we present 10 practical teaching points for educating clinical students about microaggressions and effective bystander interventions through the use of roleplay. These tips have been proposed based on the authors’ individual experiences with being on the receiving end of racial microaggressions, witnessing them in clinical settings both as a doctor and in an educator capacity, as well as awareness of current literature on this.

## Text Box 1: Summary of 10 Tips for Using Simulation for Teaching on Racial Microaggressions and Bystander Intervention


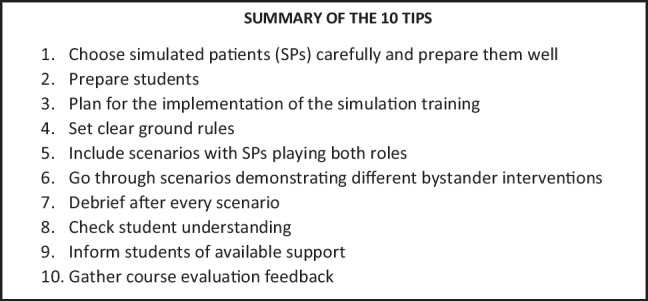


### Tip 1: Choose Simulated Patients (SPs) Carefully and Prepare Them Well

Before the SP session, make sure that the actors who will be roleplaying as either patients or healthcare professionals in the scenarios have been carefully selected. The main areas that should be focussed on are actor demographics, their understanding of racial microaggressions, and how confident they are with the scenarios.

When choosing the actors, it is important to be considerate of adequate representation in relation to race, ethnicity, age, gender, and sex. Simulation in healthcare education is an area where there have been calls for more diversity, especially when recruiting SPs. With actors usually being drawn from local communities, this can lead to a homogenous pool to choose from, resulting in educational limitations [12].

To avoid the perpetuation of stereotypes, it is advisable to avoid having one demographic as the main perpetrators or aggressors: for example, always elderly, white Caucasians. Active recruitment of actors from diverse backgrounds helps avoid misrepresentation in this way.

The SPs’ own understanding of microaggressions and bystander intervention is something that should be considered, so a briefing session before bringing in the healthcare students is beneficial. This briefing session could involve an open discussion with the simulated patients about their own biases, with an exploration of both implicit and explicit bias. In advance of the session, SPs can be directed to accepted online tools for exploring unconscious bias, particularly Implicit Association Tests (IATs) [13].

**Fig. 1:**
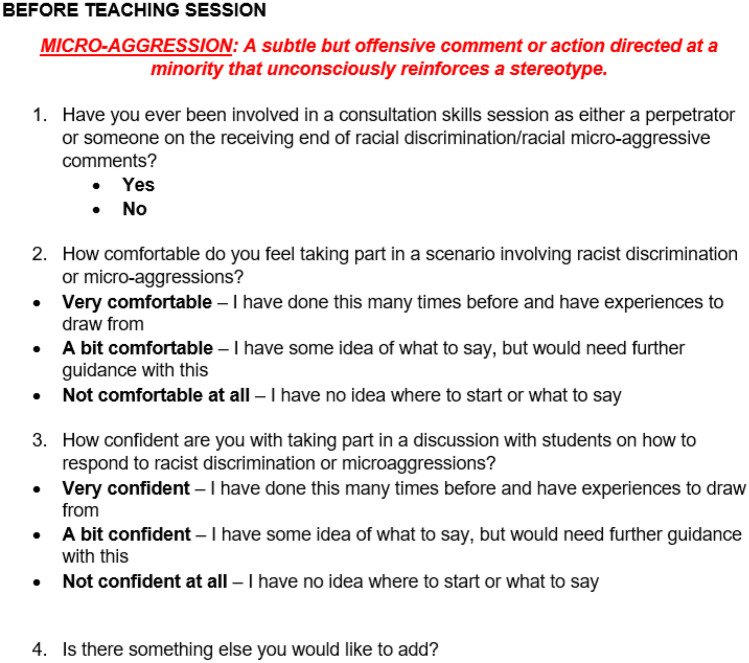
Simulated patient survey (before session)

Part of that briefing session should include a survey (Fig. 1) to gauge the actors’ understanding and how comfortable they are with the scenarios, then using this information to determine who is assigned which role. Giving clear roles (i.e. the “aggressor” who says or does racially micro-aggressive things, the “target” on the receiving end of the micro-aggressions, and “bystander” who witnesses and either does nothing or actively intervenes), and going through the scripts with the simulated patients beforehand is another way to check their confidence with the scenarios, as this will allow them to familiarise themselves with the roleplays and highlight any issues they might have with their roles.

Educators should be aware that this is a sensitive topic, and for some of the SPs, the roles they are playing might be like their own lived experiences of racism. Checking ahead of the roleplays that the SPs are happy with the scenarios is advised.

### Tip 2: Prepare the Students

Before the sessions with the SPs, introducing students to the history of racism in healthcare, as well as the theories behind racial microaggressions and unconscious bias, is a good foundation to later build on with the roleplays. Research has shown that one of the ways to become more adept at picking up on and challenging racial microaggressions is by having “content information and knowledge” about race [14], so it is important to introduce this early on, and this teaching can be given online, before the SP sessions.

This should also include introductions to different bystander intervention approaches, such as the “ABC Approach” of assessing for safety, being in a group, and caring for the target [15] as well as the “Four Ds” — direct action, distracting, delegating, and delaying [16], which they can then see in practise with the SPs. Text Boxes 2 and 4 go through both approaches with Text Box 2 using an example of a racial microaggression scenario.

This preparation work correlates with the “know” part of Miller’s pyramid of clinical competence [17], as students are able to build on their knowledge first, before progressing to the “know how” and “show” stages. These come later on when they are able to recognise racial microaggressions and start putting bystander interventions into practise.

## Text Box 2: An Explanation of the Four Ds Using an Example of a Racial Microaggression Scenario


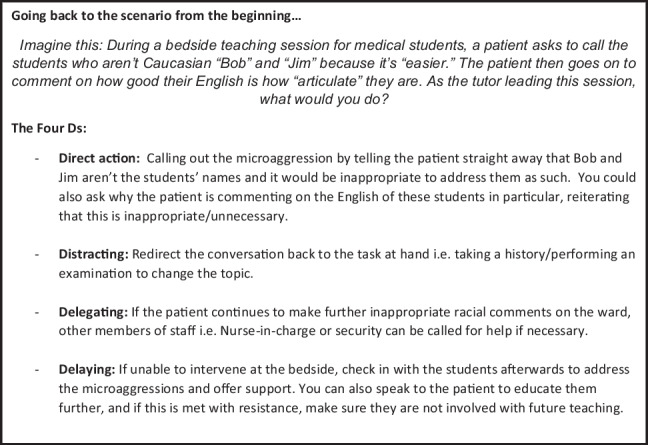


As with the SPs, students should also be prepared in advance about the content of the roleplays and how they could potentially cause distress, as the scenarios could reflect their lived experiences too.

### Tip 3: Planning for the Implementation of the Simulation Training

To maximise the productiveness of the SP session, planning for it should involve careful consideration of group size, location, and session length.

In comparison to larger groups, participation has been shown to be greater in small-group discussions, with students from different ethnic backgrounds being more likely to contribute [18]. For healthcare professions teaching (medical teaching in particular), a group size of about 8 to 12 participants has been found to be ideal, as it would enable all the students to be “regularly active” with the discussions and roleplay [19].

The physical setting for the teaching is also important. Given the sensitivity of the topic, a small seminar room would be more conducive to discussion and would be better than a formal lecture theatre.

Allocating enough time for the session is key, with planned breaks as needed for both the SPs and students. The whole session should last no more than 2 to 3 hours, which worked well with the Fisher workshops [20], as that would allow enough time to recap the online learning work, discuss the microaggressions roleplays, introduce students to the different forms of bystander intervention, and leave time for the students to put what they have learnt into practise.

### Tip 4: Set Clear Ground Rules

At the start of the session, everyone should be given the opportunity to introduce themselves to the group in order to encourage participation and student interaction. The ground rules and learning outcomes (to understand how racial microaggressions can present, and how bystander intervention works) should be outlined clearly too, so that students know what is expected of them.

Ground rules play a vital role in healthcare simulation teaching [21] and are even more necessary in teaching involving race and racism, to create a learning space that is “psychologically safe” [22]. Ground rules should cover points such as being respectful of contributions, maintaining confidentiality, and listening to each other.

To build in safety and reduce the potential for distress, it should be made clear to students that they will only be playing the role of the active bystander in the scenarios, and not the aggressor or target. Educators should emphasise that it is a safe space to share contributions, with reiteration of how confidentiality should be maintained. Students and SPs should also be given permission to time out or break out of role if necessary, as the more triggering scenarios might require this.

### Tip 5: Include Scenarios with SPs Playing Both Roles

The scenarios for the session should have the SPs in roles that show them as both the aggressors and targets of the racial microaggressions and illustrate the wide range of ways that microaggressions can present. Students should be encouraged to note what the microaggressions in the scenarios were (examples of these can be seen in Text Box 3), to prompt further discussion later. When introducing new concepts in clinical teaching, such observation works well as an initial learning step: combined with active note-taking. It has been found to be an effective teaching method [23].

As SP-based training and simulation can be expensive educational methods, recording the video scenarios of microaggressions for students to do the note taking and observation can be a cost-effective way of delivering this activity, as simulated patients would not be required to be present for this part, and sometimes can’t be present in person for every session. Another benefit of this would be for the students, as watching the videos would allow them to improve their cognitive skills through the identification of microaggressions for further discussion [24].

## Text Box 3: Examples of Racial Microaggressions


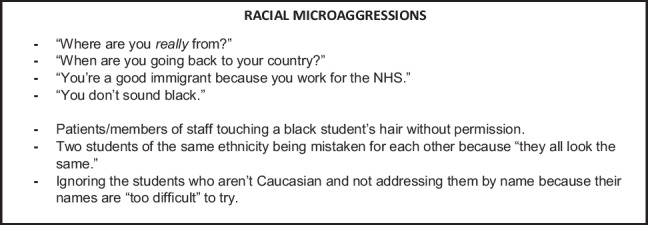


### Tip 6: Go Through Scenarios Demonstrating Different Bystander Interventions

With the pre-set online work that the students should have completed before the session, they would have already been introduced to the theory behind different bystander intervention approaches, so this would be a good opportunity to review it and practise. The “ABC approach” [14] and “4 Ds” [15] could be shown through roleplays to explain them further, with the students being encouraged to point out the part(s) of each approach being demonstrated by the actors.

### Tip 7: Debrief After Every Scenario

Debriefing has a long history of use as part of simulation and is usually a discussion with participants reflecting on scenarios, with questions exploring what happened, how it made them feel, what they would have liked to do differently, and what they would like to see happen next [25]. It encourages “reflection-on-action”, a central part of reflective practise [26], and experiential learning [27].

There are various methods for debriefing. It can happen either during or after the simulation, be instructor facilitated, or self-guided by participants, and there is the option to use video playback to assist. A systematic review by Jones and Lapkin [28] on the effectiveness of each debriefing method in health profession education concluded that although debriefing is a vital part of simulation, there is no outcome difference with video playback, so having video facilities is not essential.

For teaching on racial microaggressions and bystander intervention, debriefing could be used as an opportunity to invite the participants to share any similar experiences they might have, if they feel comfortable doing so. The constructivism approach to learning is based on the idea of peer groups teaching each other, as some will be more knowledgeable than others due to the diversity of their backgrounds and cultures — the so-called more knowledgeable others (MKOs) [29].

## Text Box 4: An Explanation of the ABC Approach in Practise


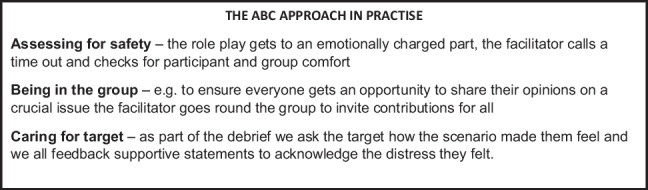


### Tip 8: Check Student Understanding

Continuous review of student understanding throughout the session is important, and one way of doing this is by having them to participate as the bystander in the roleplays. Participation in the roleplays not only allows the students to be more engaged in the session, but also gives them opportunities to practise the new bystander intervention skills they have learnt. Additionally, it would give students the opportunity to get formative feedback on their communication skills, which is an important part of ensuring effective learning in healthcare education [30].

### Tip 9: Inform Students of Available Support

Preparation for the session should involve making sure that educators are up to date and knowledgeable about support available for students in their institution. Some of them might have experienced racial microaggressions or discrimination during their training from colleagues, other healthcare professionals, or patients. Educators should signpost students to reporting and escalation procedures, as well as where to go to for additional pastoral care. From designated staff members to online reporting mechanisms, many educational and healthcare institutions have different ways to raise concerns and provide support and guidance for both staff and students, so these should always be emphasised. Educators have a duty of care to the students involved in their teaching sessions and should be prepared to mitigate harm to and support them as needed [31].

### Tip 10: Gather Course Evaluation Feedback

**Fig. 2:**
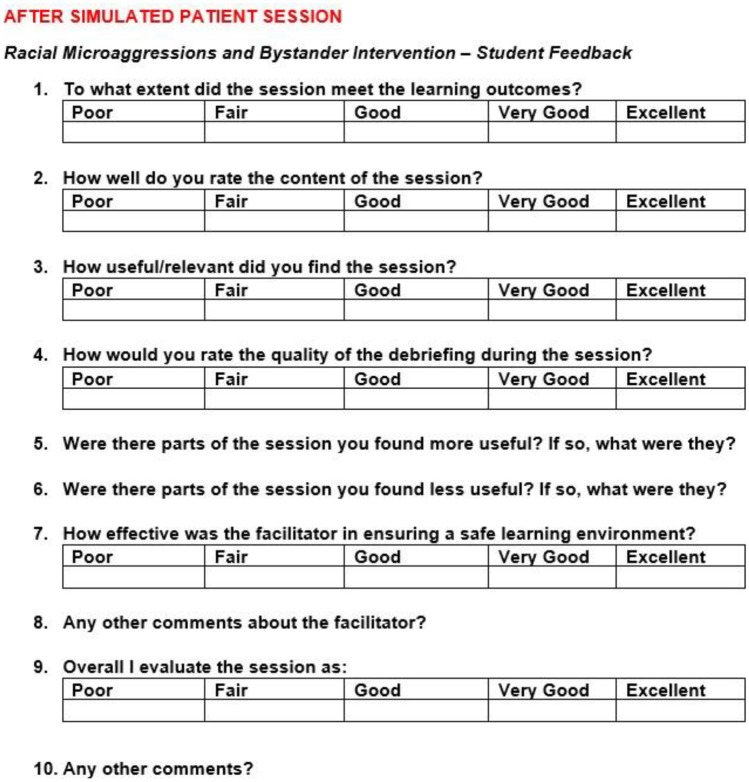
Student feedback survey (after session)

After the simulation session, questionnaires (Fig. 2) can be used to collect evaluation feedback. Giving participants the option to submit anonymously might encourage more honesty. It could be argued that with the cover of anonymity, there is a risk of students giving unhelpful, offensive, or unprofessional feedback. However, Tucker [32] investigated the number of abusive or unprofessional anonymous comments from students in online surveys at an Australian university, and results suggested that most students do not abuse this privilege.

## Conclusion

There have been calls in recent years for healthcare education to be more inclusive and representative of current issues — the COVID-19 pandemic and resurgence of the Black Lives Matter movement have really highlighted gaps in the curriculum. We need more education about racial inequality within healthcare. Due to systemic and institutional racism that dates back centuries, health inequalities disproportionately affect ethnic minorities, and this includes healthcare professionals as well as patients [33].

Dismantling ingrained beliefs and attitudes is no easy feat, but this crucial work needs to begin early in the curriculum. Teaching students about unconscious bias, racial microaggressions, bystander intervention, and the structural roots and history of racism in healthcare is a good start — the students of today will be the healthcare workers, decisions makers, and teachers of tomorrow. So using a step-by-step method with simulation as an educational tool to address this issue, whilst also exploring the viewpoints of simulated patients, students, and facilitators on how effective this is, is one way to go about it.

## Data Availability

All relevant material has been included in the manuscript.
